# Effect of social capital, social support and social network formation on the quality of life of American adults during COVID-19

**DOI:** 10.1038/s41598-024-52820-y

**Published:** 2024-02-01

**Authors:** Ebenezer Larnyo, Sharon Tettegah, Brianna Griffin, Jonathan Aseye Nutakor, Natasha Preece, Stephen Addai-Dansoh, Natalia Dubon, Senyuan Liu

**Affiliations:** 1 Center for Black Studies Research, University of California, Santa Barbara, Santa Barbara, CA 93106 USA; 2 University of California, Santa Barbara, Santa Barbara, CA 93106 USA; 3 Department of Health Policy and Management, School of Management, Jiangsu University, 301 Xuefu Road, Zhenjiang, Jiangsu Province China

**Keywords:** Geriatrics, Public health, Quality of life, Health care

## Abstract

This study aims to evaluate the effect of social capital (SC), social support (SS), and social network formation (SNF) on the quality of life of American adults during COVID-19. Using a probability sample of American adults aged 49+, 2370 respondents were selected from the National Social Life Health and Aging Project (NSHAP) dataset for analysis using an integrated partial least squares based on structural equation modeling (PLS-SEM)-K-fold cross-validation approach. The analysis showed that social capital assessed using civic engagement, social cohesion, socioeconomic status (SES), social support, and social network formation were significantly and positively associated with American adults’ quality of life during the COVID-19 pandemic. Furthermore, the results showed that using the PLS-SEM and K-fold cross-validation approach produced a medium predictive power of the overall model, confirming the importance of SC, SS, and SNF in predicting quality of life-outcomes. These findings suggest that efforts to promote the well-being of American adults, especially older adults, during the pandemic should focus on strengthening social capital, social support and social network formation.

## Introduction

Since the outbreak of COVID-19, public health officials and other auxiliary institutions have implemented lockdowns and other public health measures to prevent the spread of the virus^1^. These measures aimed to discourage large gatherings and non-essential travel and encourage staying home. Massive lockdowns meant that movement was restricted, altering people's living situations physically and socially, which presented unique issues of social isolation and a lack of connection because they were physically cut off from family and friends.

In the United States, although efforts to monitor and contain the spread of COVID-19 have received substantial national attention, literature has painted gloomy pictures of the devastating effect COVID-19 has had on individuals' social and psychological well-being^2^. The increase of COVID-19 infections and re-infections, coupled with the mutation of the virus strain and physical distancing regulations, have increased social isolation and loneliness, negatively impacting the health and well-being of many American adults, particularly, older adults, a population that is highly susceptible to social isolation. A study by Pierce et al., 2020 has documented the substantial mental health toll of the COVID-19 pandemic, exacerbated by social isolation and the disruption of social networks^3^

As a critical social determinant of health, social capital plays a protective role in the quality of life, both physical and mental health, of adults, primarily the elderly^4,5^. Researchers, over the years, have classified social capital broadly into three important aspects: civic engagement, economic connectedness, and social cohesion. Others have also extended the categorization of social capital to encompass social networks and support^4,6^. Civic engagement refers to a person’s level of involvement in their communities and their rate of volunteering and supporting other social causes^7^, while economic connectedness refers to a person’s connection based on income classification or socioeconomic status^8^. According to Corvo and De Caro, 2019^9^, social cohesiveness is the strength of relationships an individual has within a community or among friends and family groups^9^, whereas social networks represent the people in an individual's life and their interactions. Social support is direct assistance received by an individual through various social ties. Unfortunately, the pandemic has taken away the traditional avenues for assessing these essential social determinants of health, which might have consequences for the quality of life of older adults.

According to literature, having adequate social support and social networks can protect against some of the harmful consequences of other social determinants of health, such as poverty^4,10^, and a reduction in the susceptibility of persons at the bottom of the social gradient^11^. Other observational studies have also shown that social capital components are a significant protective factor for mental and physical health and mortality, with an effect equivalent to quitting smoking^12,13^. Empirical studies have demonstrated how specific social capital dimensions and orientations are associated with distinct health disparities and health inequities^12,14^. For instance, social capital interventions, particularly those that encourage social support and involvement, have a tendency to improve the overall quality of life, reduce loneliness, promote healthy lifestyles, or improve self-management of chronic illness, especially among older adults^15^. During the COVID-19 pandemic, social capital has also been recognized as a vital coping mechanism, as illustrated by studies of community volunteers leveraging social capital in China^16^.

Studies have also demonstrated a strong association between civic engagement and better health outcomes through building social capital^17–19^. For example, researchers discovered that civic organization members were more likely to be physically active^20,21^. In addition, membership in civic organizations broadened participants' social networks, making them more aware of the possibility of being physically engaged in their neighborhood^18^. Engaging in meaningful civic activities can also help build a sense of purpose, which may encourage individuals to continue participating in civic life. A multi-level analysis study of 44 countries (including the United States) also found that voting in elections or registering people to vote significantly influenced the health of such individuals^17^. In that study, the researchers observed that higher self-reported health was associated with voter involvement even after adjusting for individual and national variables^17^. During the wake of COVID-19, the United States saw an uptick in incidents of racial and ethnic discrimination, leading to massive solidarity across racial and ethnic divides, thereby creating avenues for civic engagement by older adults. However, to what extent these involvements culminated in an improvement in their health-related quality of life during COVID-19 largely remains unknown.

Socioeconomic status (SES) has long been shown to be a critical predictor for improving health outcomes and instrumental in shaping social structures regarding income inequality and economic opportunity^6,22^. Many theoretical studies have demonstrated how those with higher economic status may benefit from knowledge transmission, influence expectations, and give mentoring or job recommendations^6,22,23^. Others have also enumerated the importance of social capital in affecting the economic outcomes of entire communities. Studies have shown that societies with more social capital have higher incomes, are less corrupt, are healthier, and perform better^24^. With higher and better SES and economic connectedness, there is better health information exchange among family members and more excellent self-rated overall health^4,25^. However, the pandemic has exacerbated socioeconomic disparities impacting health behaviors and outcomes^26^. While studies have shown an association between economic connectedness and improvement in health, the economic effect of COVID-19 continues to disproportionately affect specific communities^27^, which can consequently lower their overall quality of life. For example, lower-income persons, and Black folks, are more likely than others to have gone into debt or delayed paying expenses to make up for missed earnings or salaries. In addition, Hispanic and Asian Americans are the most likely to report that they or someone in their home has lost a job or had their wages reduced since the epidemic began in February 2020^27^. This means that adults and older adults who rely on family members for sustenance will no longer receive those benefits, affecting their social capital and ultimately impacting the quality of life of these older adults.

Studies have shown that social cohesion significantly improves mental health^28,29^. Research by Cramm et al. 2012^28^ observed that the higher the level of social cohesiveness in a neighborhood, the more resources community members have access to through relationships with other individuals in the neighborhood, which improves mental health^28^. An increasing amount of research also indicates that those with higher levels of social cohesion are more likely to participate in healthier activities and have better mental and physical health. Several clarifications have been proposed on why social capital, in the form of social cohesion and social networks, may benefit health and longevity^13,30^. Social cohesion and integration into social networks might help boost self-esteem and coping skills in challenging situations such as pandemics.

Furthermore, social cohesion can promote greater empowerment and accountability. However, at the peak of the pandemic, several individuals, especially older adults, experienced increased loneliness and isolation, eliminating a critical social capital indicator such as the traditional social cohesion approach through neighborhood gatherings and other physical avenues. Thanks to the advent of technology, individuals who need to maintain social cohesion resorted to sending emails, text messaging, social media, phone calls, and other technological methods to connect with their communities, families, and friends. Whether the use of technology to replace the traditional means of acquiring social cohesion to boost the well-being and quality of life of adults and older adults during COVID-19 produced the same outcome as the conventional mode, to a large extent, remains unknown.

There is evidence that social networking allows individuals to become part of the social structure to which they belong and impacts their health^31^. Having fewer social interactions raises the risk of death from cardiovascular disease, accidents, and suicide, as well as total mortality^32^. Additionally, several studies have looked into the impact of social networks on people's mental health. For example, social relationships lower the incidence of sadness and enhance self-confidence for older adults^33^. There is also evidence that weak social networks are associated with: lower self-rated health^34^ and a worse condition of the mental and physical components of health-related quality of life in women^35,36^.

Although there is a wealth of research on the individual association between social capital, support, and mental and physical health, few studies have examined the combined effect of these factors, especially during the COVID-19 pandemic. The pandemic disrupted traditional avenues for social connection, leaving many adults, especially older adults, to rely on technology to access social capital, networks, and support. This raises the novel question of how effective technology can be in providing these essential resources and how that translates into impacting the quality of life of adult Americans. In addition, how the involvement and advocacy in racial discrimination and inequity campaigns in America can enhance civic engagement and improve the overall quality of life of adults during a pandemic, to a large extent remains unknown. Thus, to address these gaps, this study relied on a nationally representative cross-sectional sample, to investigate the impact of social capital, network, and social support on the quality of life of American adults and older adults during COVID-19. The findings from this study could be used to develop interventions to promote social capital, social support, and social network formation and improve the quality of life of adults and older adults during the pandemic and beyond.

## Methods

### Sample, sampling procedure, and data collection

Using a probability sample of American adults and older adults aged 49+, 2370 respondents were selected from the National Social Life Health and Aging Project (NSHAP) dataset^37^, with African-Americans and Hispanics sampled at a higher rate. Questionnaires for the study were distributed via a web survey, a phone interview, or a paper-and-pencil mail-back instrument. Participants were initially interviewed in Round 3 (R3) (2015–2016), and a supplementary interview was conducted during the COVID-19 pandemic between September 14, 2020, and January 27, 2021.

### Measures

#### Outcome variable

##### Quality of life during COVID-19 (CoQoL)

To evaluate the quality of life of respondents in COVID-19, this study assessed both the self-reported mental and physical health of adults and older adults. Since several studies on evaluating health-related quality of life have broadly categorized two key components, that is, mental health and physical health^38,39^, this study also relied on these two groupings. Self-rated mental health was measured by asking respondents to rate their overall mental health on a 5-Likert scale, with 5 representing excellent, three as "good," and one as "poor" mental health. Additionally, respondents were asked to report their physical health on a scale of 1 to 5, with 1 depicting poor physical health and five as "excellent" physical health.

#### Independent variables

##### Social capital (SC)

*Civic engagement (CE):* Civic engagement, or participation in activities that contribute to a community’s well-being^40^, can positively impact the quality of life of adults and older adults during the COVID-19 pandemic. Civic engagement can take many forms, such as volunteering, participating in community organizations, or advocating for issues of importance to the community. For older adults, civic engagement can provide a sense of purpose and meaning, as well as a sense of belonging and connection to the community. It can also have physical and mental health benefits^41–43^, providing opportunities for social interaction and helping reduce feelings of loneliness and isolation. During the COVID-19 pandemic, civic engagement may need to be adapted to comply with social distancing and other public health measures. However, there are still many ways for individuals to engage in their communities, such as through virtual volunteering or participating in online community events. Overall, the benefits of civic engagement for adults and older adults during the COVID-19 pandemic may include improved mental and physical health, a sense of belonging and connection to the community, and a sense of purpose and meaning.

To assess civic engagement, respondents were asked to determine the frequency of contacting representatives, signing petitions, or donating to address racial justice issues, such as the death of George Floyd or Black Lives Matter (BLM). Respondents were also asked to provide how often they had conversations with family or friends about race and racial justice, posted or shared content about racial justice on social media, or took other action regarding racial justice or BLM, using a 3-Likert scale with 0 as “never,” 1 as “sometimes” and two as “often.” This study, therefore, posits that:

###### H1

CE, as a component of SC, has a significantly positive impact on the CoQoL of adults and older adults during COVID-19.

*Social cohesion (SCoh):* Social cohesion, or the sense of belonging and connection within a community, can positively affect the quality of life of adults during the COVID-19 pandemic. Social cohesion can be promoted through volunteering, participating in community events, and interacting with neighbors and other community members. For older adults, social cohesion can provide a sense of belonging and purpose and help reduce feelings of loneliness and isolation. It can also benefit physical and mental health, as social connections and relationships are essential to overall well-being. During the COVID-19 pandemic, social cohesion may be challenged by the need to maintain physical distance and limit in-person interactions. However, there are still many ways for adults, especially older ones, to keep social connections, such as through virtual social events or regular phone or video calls with loved ones. Overall, the benefits of social cohesion for adults during the COVID-19 pandemic may include improved mental and physical health, a sense of belonging and connection to the community, and a sense of purpose and meaning. Social cohesion was evaluated by how often respondents used email, texts, social media, phone calls, and video calls to connect with their family and friends since the start of COVID-19. Based on the above literature, this study posits that:

###### H2

SCoh, a component of SC, has a significantly positive impact on the CoQoL of adults and older adults during COVID-19.

*Socioeconomic status (SES):* Socioeconomic status (SES) can significantly impact the quality of life of adults during the COVID-19 pandemic. Studies have found education and income to be among the key predictors of health among older adults^44,45^. More significantly, these studies have identified a strong positive association between higher education and better cognition^44–46^. However, adults and older adults with lower SES may be more vulnerable to the adverse effects of the pandemic, as they may have less access to healthcare, financial resources, and social support. Individuals, especially older adults with lower SES, may be more likely to experience economic insecurity and material hardship during the pandemic, as they may have limited savings or other resources to fall back on in the event of a loss of income. They may also need more access to quality healthcare, making it more challenging to manage chronic conditions or access necessary medical treatment. In addition, adults and older adults with lower SES may be more vulnerable to social isolation and loneliness, as they may have fewer social connections and support networks to rely on during the pandemic. This can have adverse effects on mental health and overall well-being. Overall, the impact of SES on the quality of life of adults and older adults during the COVID-19 pandemic may vary. Still, those with lower SES may be more vulnerable to negative impacts such as financial insecurity, limited access to healthcare, and social isolation.

Socioeconomic status was evaluated using the income classification (how well an individual's income met their basic expenses) and the educational level. Income was categorized into low, lower, middle, and upper income. Similarly, education was grouped into four classes: less than high school, high school or equivalence, vocational/associate/some college, and bachelor's degree or higher. Including these indicators is relevant in appreciating the role of SES on the quality of life of adults and older adults during COVID-19. This study, therefore, hypothesized that:

###### H3

SES, as a component of SC, has a significantly positive impact on the CoQoL of adults and older adults during COVID-19.

##### Social support (SS)

This study evaluated both tangible and emotional support received by individuals during the pandemic to assess social support. Tangible support was measured by determining the level of help an individual received in performing everyday tasks. In contrast, emotional support was measured by evaluating whether an individual received advice, comfort, love, or other forms of emotional support. Based on these questions, this study posits that:

###### H4

SS has a significantly positive impact on the CoQoL of adults and older adults during COVID-19.

##### Social network formation (SNF)

To determine the extent to which social network formation impacts the quality of life of adults and older adults, this study examined the frequency of contact (FoC) with family and friends and the relationship quality (RQ). Respondents rated the quality of their relationship with their partners as better, worse, or the same as before the COVID-19 pandemic and rated their relationship happiness on a scale of 1–7 with 1, “very unhappy,” and 7, “very happy.” How often respondents had phone calls and in-person visits with family was used to examine the frequency of contact during COVID-19. This study hypothesizes that:

###### H5a

RQ, as an SNF component, has a significantly positive impact on the CoQoL of adults and older adults during COVID-19.

###### H5b

FoC, as an SNF component, has a significantly positive impact on the CoQoL of adults and older adults during COVID-19.

**Figure 1:**
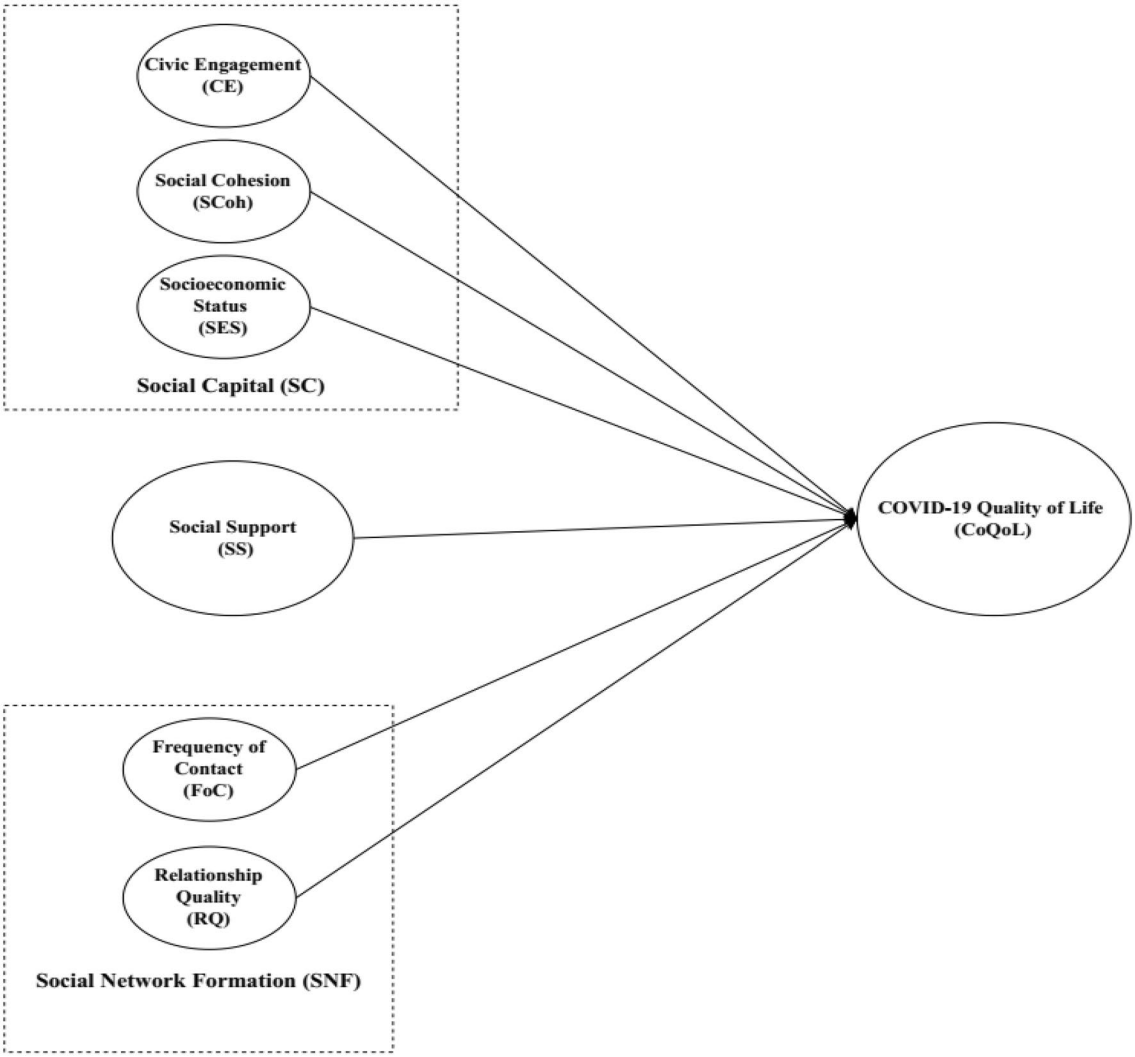
Hypothesized Framework.

Based on the hypothesis developed above, a framework was developed indicating all the interactions between the variables, as shown in Fig. 1.

### Statistical analysis

Due to its robust estimates and statistical power^38,47^, partial least squares (PLS) based on structural equation modeling (SEM) was employed to test and validate the hypothesized model. The choice of PLS-SEM, beyond those alluded to above, is well-suited to the research objectives of this study, which aims to investigate the impact of social capital, network, and social support on the quality of life of American adults during COVID-19. PLS-SEM is particularly well-suited for exploratory and predictive research^48–51^, where the focus is on understanding relationships between constructs and predicting outcomes^48–51^. Additionally, PLS-SEM is an appropriate choice for analyzing the cross-sectional data^49,52^ as used in this study. The model's validity was determined by analyzing the measurement and structural models.

The model-building process began with data preparation. This involved cleaning the data, removing any outliers, and performing imputation for the missing values. Next, the measurement model was assessed by testing the validity of the constructs and indicators in the model. Since the model in this study is formative, the measurement model was evaluated using convergent validity (redundancy analysis), VIF, the statistical significance of weights (t-statistics or *p*-values), and the relevance of indicators. According to Hair et al.^53^, correlation values of redundancy analysis must be ≥ 0.70 to be considered acceptable for single-item constructs, while VIF ≥ 5 indicates probable collinearity problems, ranging between 3 and 5 as a possibility for collinearity problems and < 3 indicating no collinearity problems^53,54^. For the significance of the outer weights, t-statistics values have to be 2.576 (1% CI), 1.96 (5% CI), and 1.645 (10% CI), respectively, to be considered significant^54^. The relevance of the indicators with a significant weight is determined based on the size of the indicator weights, with larger standardized indicator values closer to + 1 (− 1), suggesting a strong relative contribution to the constructs. If a confidence interval does not contain a zero value, the weight is statistically significant, and the indicator can be kept. On the other hand, if an indicator weight's confidence interval contains 0, it implies that the weight is not statistically significant (assuming the provided significance threshold, e.g., 5%). In this case, the indicator should be eliminated from the measurement model^54^.

Once the measurement model was deemed to be satisfactory, the structural model was assessed by examining the hypothesized relationships between the constructs. The structural model was evaluated using the variance inflation factors (VIF), path coefficient (β), t-statistics, *p*-values, and (*Q*^2^ predict)^54^. Model fit analysis was also performed using the Standardized Root Mean Square Residual (SRMR), discrepancy function (d_ULS and d_G), chi-square statistic, and the Normed Fit Index (NFI). SRMR < 0.08 is considered acceptable^55^, while the closer the NFI is to 1, the better the fit. NFI values above 0.9 usually represent an acceptable fit^56^. K-fold cross-validation^57^ was used to assess the model's overall predictive performance using PLSPredict^58^ with ten folds and ten iterations. A number of different models were built and tested before the final model was chosen. The final model was chosen because it had the best fit for the data and the most theoretically sound relationships. The PLS-SEM analysis was performed using SmartPLS 4.0^59^.

### Handling missing data

To account for the missing data in the dataset, Multivariate Imputation by Chained Equations (MICE)^60^ was used. In all, five imputations (m = 5) were done in twenty iterations (maxit = 20) using the Predictive Mean Matching (pmm) technique^61^ in R.

### Ethical approval and consent to participate

All protocols for the NSHAP were approved by the National Opinion Research Center (NORC) at the University of Chicago (Protocol Number: 14.06.01) and carried out in accordance with the Declaration of Helsinki and other essential guidelines and ethical review regulations that applies to conducting studies involving human participants. The approval also covered all procedures through which written informed consent was obtained from each participant, including them being informed about the objective of the study, and guarantee of anonymity. Confidential records of participants’ consent were maintained by NORC and thus, this current study did not include any confidential records of participants. Furthermore, informed consents for participating in the study were obtained from each participant.

### Consent to participate

Informed consent was obtained from all respondents involved in the study.

## Results

### Descriptive statistics

Results of the respondent’s demography are shown in Table 1. Of the total respondents, 735 were aged 60–69 years. The number of females was 1318, while males were 1052. Furthermore, the most observed category for the level of education was vocational/some college/associate and the most observed category income level was middle-income level.

**Table 1:** Sample demographic characteristics.

	Freq	%
Age		
49–59	639	26.96
60–69	735	31.01
70–79	731	30.84
80–90	265	11.18
Gender		
Male	1052	44.39
Female	1318	55.61
Marital status		
Married	1671	70.51
Living with a partner	62	2.62
Separated	30	1.27
Divorced	250	10.55
Widowed	268	11.31
Never married	89	3.76
Education		
Less than high school	231	9.75
High school/equivalence	504	21.27
Vocational/some college/associate	833	35.15
Bachelors or more	802	33.84
Income level		
Low	55	2.32
Lower	173	7.30
Middle	1127	47.55
Upper income	1015	42.83

### Evaluation of measurement model

Collinearity, outer weights, t-statistics, and *p*-values were used to evaluate the measurement model as shown in Table 2. Results revealed that almost all constructs had outer weights higher than 0.5, except for CE2, SCoh1, and FoC1; however, CE2 and SCoh1 were statistically significant. Furthermore, all constructs had VIF < 3 suggesting no collinearity problems.

**Table 2:** Measurement Model.

Constructs	Notation	Convergent validity	Collinearity	Statistical significance of weights	
		Outer weights	VIF	T-statistics	*P*-value
Social capital					
Civic engagement	CE1	0.845	1.536	13.934	0.000
	CE2	0.233	1.536	2.863	0.004
Social cohesion	SCoh1	0.424	1.715	3.029	0.002
	SCoh2	0.672	1.715	5.264	0.000
Socioeconomic status	EDUCATION	0.626	1.091	10.905	0.000
	INCOME LEVEL	0.620	1.091	10.845	0.000
Social support					
Tangible help	SS1	0.533	1.057	3.705	0.000
Emotional support	SS2	0.732	1.057	6.153	0.000
Social network formation					
Relationship quality	RQ1	0.800	1.008	9.705	0.000
	RQ2	0.532	1.008	4.519	0.000
Frequency of contact	FoC1	0.152	1.119	0.285	0.776
	FoC2	0.940	1.119	2.373	0.018
Quality of life					
Mental health	MNTLTH	0.530	1.141	8.375	0.000
Physical health	PHYSHLTH	0.682	1.141	12.292	0.000

### Structural model, hypothesis testing, and robustness check

**Figure 2:**
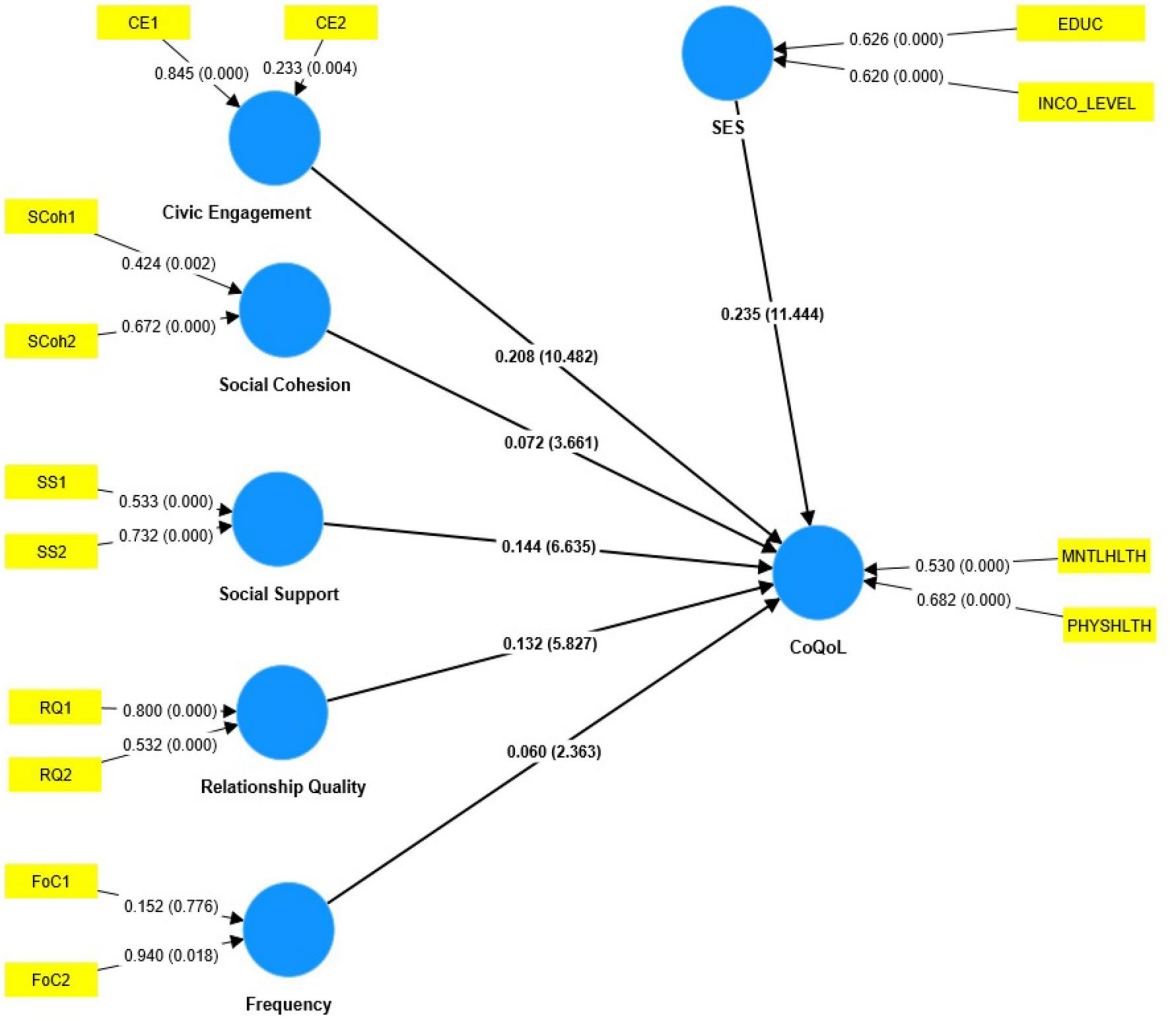
Structural Model with bootstrapped results.

Results of the structural model are shown in Table 3 and Figure 2, respectively.

**Table 3:** Structural Model and Robustness Check.

Hypothesis	Path	Path coefficient (β)	t-Statistics	*P* value	Hypothesis supported or not
H1	CE → CoQoL	0.209	10.517	0.000	Supported
H2	SCoh → CoQoL	0.072	3.658	0.000	Supported
H3	SES → CoQoL	0.235	11.377	0.000	Supported
H4	SS → CoQoL	0.143	6.672	0.000	Supported
H5a	RQ → CoQoL	0.132	5.846	0.000	Supported
H5b	FoC → CoQoL	0.059	2.881	0.004	Supported

Fit Indices	Saturated Model	Estimated Model			
Model Fit					
SRMR	0.043	0.043			
d_ULS	0.192	0.192			
d_G	0.043	0.043			
Chi-square	547.326	547.326			
NFI	0.888	0.888			

Constructs	*Q*^*2*^ Predict	PLSPredict			
		PLS-SEM RMSE	LM RMSE		
Result of Predictive Relevance (*Q*^2^* Predict*) and K-fold Cross-Validation (tenfold, ten iterations)					
Quality of Life					
MNTLHLTH	0.112	0.90	0.90		
PHYSHLTH	0.144	0.90	0.90

Results of the structural model revealed a statistically significant relationship between all the constructs, thus, supporting hypotheses H1, H2, H3, H4, H5a, and H5b, respectively. The summary of the fit indexes showed SRMR less than 0.08 and NFI approximately equal to 0.90, thus, suggesting model fit, as shown in Table 3. Furthermore, *Q*^2^ predict values were greater than zero indicating the model outperformed the most naïve benchmark. At the same time, the K-fold cross-validation result for both the PLS model and LM model produced an RMSE of 0.90, suggesting the overall model had a medium predictive power.

## Discussion

This study evaluated the impact of social capital, social support, and social network formation on American adults’ quality of life during the COVID-19 pandemic. The study found a significant positive relationship between civic engagement, SES, and social cohesion, which were used to measure social capital and quality of life during COVID-19. These findings are consistent with previous studies that have established an association between social capital and quality of life pre-COVID-19^62–65^.

Research has shown that adults, especially older adults who are civically engaged, tend to have better physical and mental health outcomes^41–43^, as well as a greater sense of purpose and meaning in their lives. Civic engagement can include, volunteering, participating in community events, and in the case of this study, supporting racial justice and equity. During the pandemic, civically engaged individuals were more likely to have access to information and resources that helped them cope with the pandemic's challenges. For example, adult and older adults involved in community organization initiatives to bridge the racial inequality gap in healthcare and other social issues were more likely to know about local resources and support available. Additionally, civically engaged individuals were more likely to be connected to their community, which can provide a sense of belonging and social support.

Social cohesion and SES significantly correlated with better quality of life during the pandemic. These findings are in line with previous studies^66–68^. Research has shown that older adults who live in communities with high levels of social cohesion tend to have better physical and mental health outcomes^69,70^. This is likely because social cohesion can provide adults, especially those advanced in age, with a sense of belonging and support, which can mitigate the adverse effects of stress and isolation. Socioeconomic status (SES), a measure of an individual's economic and social position in society, is also an important factor in adults' quality of life during the COVID-19 pandemic. This status can be influenced by income, education, and occupation. During the pandemic, many people lost their sources of livelihood, and for people of color, that translates into issues of food insecurity, loss of home, shelter, among other things. Research has shown that individuals from lower SES backgrounds are more likely to experience adverse physical and mental health outcomes during the pandemic^71^. This is likely because individuals from lower SES backgrounds may need more access to resources and support, making it more difficult for them to cope with the challenges of the pandemic. Thankfully, due to the introduction of many pro-poor interventions during the pandemic^72^, many people received some financial relief due to the heightened demand for social justice and equity; hence, this could explain the positive association between SES and quality of life in this study. Also, individuals had sufficient income and financial reserves pre-pandemic which they fell back on to provide for their sustenance, thus, causing a positive impact on their overall quality of life.

A significant positive association was observed between social network formation assessed by the frequency of contact and the quality of relationships among older adults and their quality of life. Previous studies have shown that social network formation is positively associated with the quality of life of adults and older adults^30,73,74^. For example, a study by Berkman and Glass^30^ found that older adults with more extensive and diverse social networks tended to have better physical and mental health outcomes^30^. Similarly, a study by Vos et al.^74^ found that older adults who reported a higher level of social network formation were less likely to experience adverse mental health outcomes during the COVID-19 pandemic^74^. Furthermore, a study by Charles et al.^75^ found that older adults who reported a higher level of relationship quality were less likely to experience adverse mental health outcomes^75^. While there is no definite evidence that social network influences the incidence of illness, it appears that it does enhance the likelihood of a favorable prognosis in the case of heart ischemia and cerebrovascular disease^76^. This is likely because social networks can provide individuals access to a broader range of resources and support, including information, resources, and practical assistance, thus positively impacting their quality of life.

Finally, a significant positive relationship was established between social support and the quality of life of adults and older adults during the pandemic in America. Research has shown that older adults with a strong social support network tend to have better physical and mental health outcomes during times of stress^73,77,78^, such as during a pandemic. This is most likely because social support can help mitigate the adverse effects of stress and isolation, which can be especially severe in older adults. This study observed that adults and older adults who had the financial means to get physical or tangible help could do so even if their immediate family members or friends could not provide them with finances. Others also alluded to the fact that their family and friends could offer emotional support to them through various technologies and social media, consequently serving as a protective factor against their stress and anxiety, leading to a better self-rated quality of life.

## Conclusion

This study examined the effects of social capital, social support, and social network formation on American adults' quality of life during COVID-19. The findings of this study indicate that social capital, social support, and social network formation positively impact the quality of life of American adults during the COVID-19 pandemic. Furthermore, the findings of this study underscore the importance of taking individual and community-level factors into account when developing interventions and policies aimed at improving the well-being of Americans, particularly for older adults during the COVID-19 pandemic and beyond. At the individual level, it is imperative to encourage adults to participate in civic activities, such as volunteering, participating in community events, and supporting racial justice and equity causes. This can help individuals stay connected to their community and find purpose and meaning in their lives. Steps should also be taken to address the social and economic determinants of health, such as poverty, income inequality, and lack of access to healthcare and other essential resources, as these will help to improve the overall well-being of adults, regardless of their social status. At the community level, there is a need to promote social cohesion by building strong community ties and fostering a sense of belonging among American adults. This can be done by supporting community organizations and programs that bring people together. Furthermore, it is important to ensure that adults have access to quality healthcare and other essential services, such as food, housing, and transportation, as these will help adults to meet their basic needs and live healthy and fulfilling lives. Based on the findings of this study, we also recommend the creation of age-friendly communities that are safe, accessible, and inclusive for older adults. This can be done by making modifications to public spaces and transportation systems, and by providing programs and services that are tailored to the needs of older adults.

## Limitation

Though this current study provides valuable insight into the subject matter, it is essential to note that it has some limitations that may not be generalizable to other adult populations and geographic locations.

First, the data used for this study is specific to geographical locations in the United States of America; thus, the conclusions drawn are relative to those locations. Hence, relying on this study to understand the effect of the constructs used in other jurisdictions should be done cautiously, as the situations during COVID-19 in the USA might be different in those locations.

Second, the study was conducted during the early stages of the pandemic; hence, it might not have captured the broader duration, more so as the pandemic is still active. It will therefore be essential to replicate the study in the future to examine the longer-term effects of social capital, social support, and social network formation on the quality of life of adults and older adults during the COVID-19 pandemic.

### Supplementary Information

Supplementary Tables.

## Data Availability

The National Social Life Health and Aging Project (NSHAP) Round 3 and COVID-19 Study dataset^37^ was used for this study and can be publicly obtained from https://www.icpsr.umich.edu/web/ICPSR/studies/36873.

## References

[1] Larnyo E (2021). Assessing the impact of social media use on everyday emotion in health crises: A study of international students in China during COVID-19. Healthcare.

[2] Saladino V, Algeri D, Auriemma V (2020). The psychological and social impact of Covid-19: New perspectives of well-being. Front. Psychol..

[3] Pierce M (2020). Mental health before and during the COVID-19 pandemic: A longitudinal probability sample survey of the UK population. Lancet Psychiatry.

[4] Green H (2022). Social capital and wellbeing among Australian adults’ during the COVID-19 pandemic: a qualitative study. BMC Public Health.

[5] Wilkinson, R., Marmot, M. & Organization, W.H. *Social Detearminants of Health: The Solid Facts*. 2003: World Health Organization. Regional Office for Europe.

[6] Chetty R (2022). Social capital I: Measurement and associations with economic mobility. Nature.

[7] Habib, D. R. S., Klein, L. M., Perrin, E. M., Perrin, A. J., Johnson, S. B. The Role of Primary Care in Advancing Civic Engagement and Health Equity: A Conceptual Framework. *Milbank Q.* **101**(3), 731–767. 10.1111/1468-0009.12661 (2023). 10.1111/1468-0009.12661 PMC10509514 37347445

[8] Chetty, R. *et al*. Social capital II: determinants of economic connectedness. *Nature* **608**, 122–134. 10.1038/s41586-022-04997-3 (2022). 10.1038/s41586-022-04997-3 PMC9352593 35915343

[9] Corvo E, De Caro W (2019). Social capital and social networks. Eur. J. Pub. Health.

[10] Achdut N, Refaeli T, Schwartz Tayri TM (2021). Subjective poverty, material deprivation indices and psychological distress among young adults: The mediating role of social capital and usage of online social networks. Soc. Indic. Res..

[11] Cattell V (2001). Poor people, poor places, and poor health: The mediating role of social networks and social capital. Soc. Sci. Med..

[12] Coll-Planas L (2017). Social capital interventions targeting older people and their impact on health: A systematic review. J. Epidemiol. Commun. Health.

[13] Nyqvist F (2014). Structural and Cognitive aspects of social capital and all-cause mortality: A meta-analysis of cohort studies. Soc. Indic. Res..

[14] Fujiwara T, Kawachi I (2008). A prospective study of individual-level social capital and major depression in the United States. J. Epidemiol. Commun. Health.

[15] Blancafort Alias S (2021). Promoting social capital, self-management and health literacy in older adults through a group-based intervention delivered in low-income urban areas: Results of the randomized trial AEQUALIS. BMC Public Health.

[16] Miao Q, Schwarz S, Schwarz G (2021). Responding to COVID-19: Community volunteerism and coproduction in China. World Dev..

[17] Kim S, Kim CY, You MS (2015). Civic participation and self-rated health: A cross-national multi-level analysis using the world value survey. J. Prev. Med. Public Health.

[18] Marquez B (2016). Latino civic group participation, social networks, and physical activity. Am. J. Health Behav..

[19] Putnam RD (2015). Bowling alone: America’s declining social capital. The City Reader.

[20] Zhang, W. *et al*. Increased frequency of participation in civic associations and reduced depressive symptoms: Prospective study of older Japanese survivors of the Great Eastern Japan Earthquake. *Soc. Sci. Med.* **276**, 113827. 10.1016/j.socscimed.2021.113827 (2021). 10.1016/j.socscimed.2021.113827 PMC9136530 33744732

[21] Dubowitz, T. *et al.* Chandra A. Factors related to health civic engagement: results from the 2018 National Survey of Health Attitudes to understand progress towards a Culture of Health. *BMC Public Health.* **20**(1), 635. 10.1186/s12889-020-08507-w (2020). 10.1186/s12889-020-08507-w PMC7203885 32380964

[22] Putnam RD (2016). Our Kids: The American Dream in Crisis.

[23] Ambrus A, Mobius M, Szeidl A (2014). Consumption risk-sharing in social networks. Am. Econ. Rev..

[24] Makridis, C. & Wu, C. *Ties that Bind (and Social Distance): How Social Capital Helps Communities Weather the COVID-19 Pandemic.* Available at SSRN 3592180 (2020).

[25] Lee JK, Lin L, Wu XV (2020). Social capital and health communication in Singapore: An examination of the relationships between community participation, perceived neighborliness and health communication behaviors. J. Health Commun..

[26] Guo Z (2021). Socioeconomic disparities in ehealth literacy and preventive behaviors during the COVID-19 pandemic in Hong Kong: Cross-sectional study. J. Med. Internet Res..

[27] Horowitz, J.M., Brown, A. & Minkin, R. *A Year Into the Pandemic, Long-Term Financial Impact Weighs Heavily on Many Americans *(2021).

[28] Cramm JM, van Dijk HM, Nieboer AP (2012). The importance of neighborhood social cohesion and social capital for the well being of older adults in the community. Gerontol..

[29] Mitchell CU, LaGory M (2002). Social capital and mental distress in an impoverished community. City Commun..

[30] Berkman LF, Glass T (2000). Social integration, social networks, social support, and health. Soc. Epidemiol..

[31] García EL (2005). Social network and health-related quality of life in older adults: A population-based study in Spain. Qual. Life Res..

[32] Eng PM (2002). Social ties and change in social ties in relation to subsequent total and cause-specific mortality and coronary heart disease incidence in men. Am. J. Epidemiol..

[33] Chen Y-RR, Schulz PJ (2016). The effect of information communication technology interventions on reducing social isolation in the elderly: A systematic review. J. Med. Internet Res..

[34] Charonis A (2017). Subjective social status, social network and health disparities: Empirical evidence from Greece. Int. J. Equity Health.

[35] Boehlen FH (2022). Loneliness as a gender-specific predictor of physical and mental health-related quality of life in older adults. Qual. Life Res..

[36] Rhee TG, Marottoli RA, Monin JK (2021). Diversity of social networks versus quality of social support: Which is more protective for health-related quality of life among older adults?. Prev. Med..

[37] Waite, L.J., *et al*., *National Social Life, Health, and Aging Project (NSHAP): Round 3 and COVID-19 Study, [United States], 2015-2016, 2020-2021*. 2022, Inter-university Consortium for Political and Social Research [distributor] 10.3886/ICPSR36873.v7.

[38] Larnyo E (2022). Impact of actual use behavior of healthcare wearable devices on quality of life: A cross-sectional survey of people with dementia and their caregivers in Ghana. Healthcare.

[39] Moller V (1987). Quality of Life in South Africa: Measurement and Analysis.

[40] Wray-Lake, L., DeHaan, C. R., Shubert, J., & Ryan, R. M. Examining links from civic engagement to daily well-being from a self-determination theory perspective. *J. Posit. Psychol.* **14**(2), 166–177. 10.1080/17439760.2017.1388432 (2019).

[41] Gottlieb BH, Gillespie AA (2008). Volunteerism, Health, and civic engagement among older adults. Can. J. Aging/ Revue Can Vieillissement.

[42] Lum TY, Lightfoot E (2005). The effects of volunteering on the physical and mental health of older people. Res. Aging.

[43] McDougle L (2014). Health outcomes and volunteering: The moderating role of religiosity. Soc. Indic. Res..

[44] Larnyo E (2022). Examining the impact of socioeconomic status, demographic characteristics, lifestyle and other risk factors on adults’ cognitive functioning in developing countries: An analysis of five selected WHO SAGE Wave 1 countries. Int. J. Equity Health.

[45] Koster A (2005). Socioeconomic differences in cognitive decline and the role of biomedical factors. Ann. Epidemiol..

[46] Evans DA (1993). Level of education and change in cognitive function in a community population of older persons. Ann. Epidemiol..

[47] Reinartz W, Haenlein M, Henseler J (2009). An empirical comparison of the efficacy of covariance-based and variance-based SEM. Int. J. Res. Market..

[48] Hair JF (2021). A Primer on Partial Least Squares Structural Equation Modeling (PLS-SEM).

[49] Hair JF (2017). A Primer on Partial Least Squares Structural Equation Modeling (PLS-SEM).

[50] Chin WW (1998). The partial least squares approach to structural equation modeling. Mod. Methods Bus. Res..

[51] Hair JF (2021). Partial Least Squares Structural Equation Modeling (PLS-SEM) Using R: A Workbook.

[52] Garson GD (2016). Partial Least Squares. Regression and Structural Equation Models.

[53] Hair JF, Sarstedt M, Ringle CM (2019). Rethinking some of the rethinking of partial least squares. Eur. J. Market..

[54] Hair JF, Hair JF (2021). Evaluation of formative measurement models. Partial Least Squares Structural Equation Modeling (PLS-SEM) Using R: A Workbook.

[55] Hu LT, Bentler PM (1999). Cutoff criteria for fit indexes in covariance structure analysis: Conventional criteria versus new alternatives. Struct. Equ. Mode: Multidiscip. J..

[56] Kline RB (2011). Principles and Practice of Structural Equation Modeling (3 Baskı).

[57] Jung Y (2018). Multiple predicting K-fold cross-validation for model selection. J. Nonparametric Stat..

[58] Hair JF (2019). When to use and how to report the results of PLS-SEM. Eur. Bus. Rev..

[59] Ringle CM, Wende S, Becker J-M (2022). SmartPLS 4.

[60] Van Buuren S, Oudshoorn CG (2000). Multivariate Imputation by Chained Equations.

[61] Vink G (2014). Predictive mean matching imputation of semicontinuous variables. Stat. Neerl..

[62] Hassanzadeh J (2016). Association between social capital, health-related quality of life, and mental health: A structural-equation modeling approach. Croat. Med. J..

[63] Zhong Y (2017). Association between social capital and health-related quality of life among left behind and not left behind older people in rural China. BMC Geriatr..

[64] Amegbor PM (2020). Effect of cognitive and structural social capital on depression among older adults in Ghana: A multilevel cross-sectional analysis. Arch. Gerontol. Geriatr..

[65] Gray A (2009). The social capital of older people. Ageing Soc..

[66] Jewett RL (2021). Social cohesion and community resilience during COVID-19 and pandemics: A rapid scoping review to inform the united nations research roadmap for COVID-19 recovery. Int. J. Health Serv..

[67] Zhang J, Hong L, Ma G (2022). Socioeconomic Status, peer social capital, and quality of life of high school students during COVID-19: A mediation analysis. Appl. Res. Qual. Life.

[68] Silveira S (2022). Coping with the COVID-19 pandemic: Perceived changes in psychological vulnerability, resilience and social cohesion before, during and after lockdown. Int. J. Environ. Res. Pub. Health.

[69] Li Y (2023). The role of community cohesion in older adults during the COVID-19 epidemic: Cross-sectional study. JMIR Pub. Health Surveill..

[70] Choi NG (2015). perceived social cohesion, frequency of going out, and depressive symptoms in older adults: Examination of longitudinal relationships. Gerontol. Geriatr. Med..

[71] Geranios K, Kagabo R, Kim J (2022). Impact of COVID-19 and socioeconomic status on delayed care and unemployment. Health Equity.

[72] Service, I.R. *Economic Impact Payments: What You Need to Know*. 2021 15th March, 2023. Retrieved 3 Jan 2023 from https://www.irs.gov/coronavirus/economic-impact-payments.

[73] Berkman L, Kawachi I (2001). Social ties and mental health. J. Urban Health.

[74] Vos WH (2020). Exploring the impact of social network change: Experiences of older adults ageing in place. Health Soc. Care Commun..

[75] Charles ST (2009). Now you see it, now you don’t: Age differences in affective reactivity to social tensions. Psychol. Aging.

[76] Gariepy G, Honkaniemi H, Quesnel-Vallee A (2016). Social support and protection from depression: systematic review of current findings in Western countries. Br. J. Psychiatry.

[77] Khan A, Husain A (2010). Social support as a moderator of positive psychological strengths and subjective well-being. Psychol. Rep..

[78] Eisman AB (2015). Depressive symptoms, social support, and violence exposure among urban youth: A longitudinal study of resilience. Dev. Psychol..

