# Supplementary material for: Effect of social capital, social support and social network formation on the quality of life of American adults during COVID-19
Source: Sci Rep. 2024 Feb 1;14:2647. doi: 10.1038/s41598-024-52820-y (PMC10834438; doi:10.1038/s41598-024-52820-y)
Supplement: Supplementary file 1 — Supplementary Tables. [file 41598_2024_52820_MOESM1_ESM.doc]

| **Constructs** | | **Notation** | **Convergent Validity** | **Collinearity** | **Statistical Significance of Weights** | |
| --- | --- | --- | --- | --- | --- | --- |
|  |  |  | **Outer**  **Weights** | **VIF** | **T-statistics** | **P-value** |
| Social Capital | Civic Engagement | CE1 | 0.857 | 1.536 | 20.609 | 0.000 |
|  |  | CE2 | 0.346 | 1.536 | 5.037 | 0.000 |
|  | Social Cohesion | SCoh1 | 0.401 | 1.715 | 2.875 | 0.004 |
|  |  | SCoh2 | 0.694 | 1.715 | 5.601 | 0.000 |
|  | Socioeconomic Status | EDUCATION | 0.631 | 1.091 | 11.164 | 0.000 |
|  |  | INCOME LEVEL | 0.615 | 1.091 | 10.861 | 0.000 |
| Social Support | Tangible Help | SS1 | 0.513 | 1.057 | 3.600 | 0.000 |
|  | Emotional Support | SS2 | 0.749 | 1.057 | 6.564 | 0.000 |
| Social Network Formation | Relationship Quality | RQ1 | 0.686 | 1.008 | 4.899 | 0.000 |
|  |  | RQ2 | 0.667 | 1.008 | 4.560 | 0.000 |
|  | Frequency of Contact | FoC1 | 0.087 | 1.119 | 0.257 | 0.797 |
|  |  | FoC2 | 0.981 | 1.119 | 7.251 | 0.000 |
| Quality of Life | Mental Health | MNTLTH | 0.490 | 1.141 | 8.527 | 0.000 |
|  | Physical Health | PHYSHLTH | 0.718 | 1.141 | 15.032 | 0.000 |

Measurement Model for Original Dataset without imputation

Structural Model and Robustness Check for Original Dataset without imputation

| **Hypothesis** | **Path** | ***path coefficient***  ***(β)*** | ***t*-**  **Statistics** | | ***P***  **Value** | ***Hypothesis supported or not*** |
| --- | --- | --- | --- | --- | --- | --- |
| **H1** | **CE→ CoQoL** | 0.198 | 10.139 | | 0.000 | Supported |
| **H2** | **SCoh→ CoQoL** | 0.076 | 3.808 | | 0.000 | Supported |
| **H3** | **SES → CoQoL** | 0.248 | 11.988 | | 0.000 | Supported |
| **H4** | **SS → CoQoL** | 0.139 | 6.290 | | 0.000 | Supported |
| **H5a** | **RQ →** **CoQoL** | 0.093 | 4.189 | | 0.000 | Supported |
| **H5b** | **FoC → CoQoL** | 0.032 | 1.543 | | 0.123 | Not Supported |
| **Model Fit** | | | | | | |
| **Fit Indices** | **Saturated Model** | | | **Estimated Model** | | |
| **SRMR** | 0.039 | | | 0.039 | | |
| **d_ULS** | 0.160 | | | 0.160 | | |
| **d_G** | 0.037 | | | 0.037 | | |
| **Chi-square** | 462.830 | | | 462.830 | | |
| **NFI** | 0.878 | | | 0.878 | | |
